# Reduction in magnetic resonance imaging T2 burden of disease in patients with relapsing-remitting multiple sclerosis: analysis of 48-week data from the EVIDENCE (EVidence of Interferon Dose-response: European North American Comparative Efficacy) study

**DOI:** 10.1186/1471-2377-8-11

**Published:** 2008-04-21

**Authors:** A Traboulsee, A AL-Sabbagh, R Bennett, P Chang, DKB Li, the EVIDENCE Study Group and the UBC MS/MRI Research Group

**Affiliations:** 1 Division of Neurology, Medicine, University of British Columbia, Vancouver, BC, Canada; 2 EMD Serono, Inc., Rockland, MA, USA; 3 Radiology, University of British Columbia, Vancouver, BC, Canada

## Abstract

**Background:**

The EVIDENCE (EVidence of Interferon Dose-response: European North American Comparative Efficacy) study was an international, randomized, open-label, assessor-blinded, parallel-group study assessing the efficacy and tolerability of interferon (IFN) beta-1a, 44 mcg subcutaneously (sc) three times weekly (tiw), and IFN beta-1a, 30 mcg intramuscularly (im) once weekly (qw), in patients with relapsing-remitting multiple sclerosis (RRMS). The aim of this analysis was to assess whether reductions in T2 burden of disease (BOD) were greater for patients receiving IFN beta-1a, 44 mcg sc tiw, than for those treated with IFN beta-1a, 30 mcg im qw, and to assess the impact of neutralizing antibodies (NAbs).

**Methods:**

A post-hoc analysis was performed on magnetic resonance imaging (MRI) data collected prospectively from the EVIDENCE study. The analysis included all patients with evaluable T2 MRI scans at the start of dosing and at week 48, and those who received at least one drug dose (n = 553). Lesions were identified by a radiologist blinded to treatment codes and the total volume of T2 lesions (BOD) was reported in mm^3^.

**Results:**

Both median percentage decreases and absolute reduction in BOD were greater in the IFN beta-1a, 44 mcg sc tiw, treatment group. The adjusted mean treatment difference in percentage change in BOD from baseline to week 48 showed a significant treatment benefit for patients treated with IFN beta-1a, 44 mcg sc tiw, over those treated with IFN beta-1a, 30 mcg im qw (-4.6%; standard error: 2.6%; p = 0.002). The presence of NAbs reduced the effect of IFN beta-1a 44, mcg sc tiw, on BOD, but BOD changes were still similar to those seen with IFN beta-1a, 30 mcg im qw.

**Conclusion:**

Patients with RRMS treated with IFN beta-1a, 44 mcg sc tiw, had greater reduction in T2 BOD after 48 weeks than those treated with IFN beta-1a, 30 mcg im qw, which is consistent with other clinical and MRI outcome measures in the EVIDENCE study. In patients testing positive for NAbs (NAb+) to IFN beta-1a 44 mcg sc tiw, changes in BOD were smaller than in NAb negative (NAb-) patients, but similar to those receiving IFN beta-1a, 30 mcg im qw.

## Background

Interferon (IFN) beta-1a, 44 mcg subcutaneously (sc) three times weekly (tiw), and IFN beta-1a, 30 mcg intramuscularly (im) once weekly (qw), are both licensed for the treatment of patients with relapsing forms of multiple sclerosis (MS). These two IFN beta-1a formulations were compared in the EVIDENCE (EVidence of Interferon Dose-response: European North American Comparative Efficacy) study, which was an international, randomized, open-label, assessor-blinded, parallel-group study to determine if IFN beta-1a, 44 mcg sc tiw, has greater efficacy on clinical and magnetic resonance imaging (MRI) outcomes to that of IFN beta-1a, 30 mcg im qw, in patients with relapsing-remitting MS (RRMS). Patients treated with IFN beta-1a, 44 mcg sc tiw, had a significantly higher odds ratio for remaining relapse free at 24 weeks (p = 0.0005), at 48 weeks (p = 0.009) and over an average of 64 weeks (p = 0.023), compared with patients treated with IFN beta-1a, 30 mcg im qw [1-3]. In addition, at the same time points, new activity on MRI was significantly lower in patients receiving IFN beta-1a, 44 mcg sc tiw, than in those receiving IFN beta-1a, 30 mcg im qw: reductions were seen in gadolinium (Gd)-enhancing lesions, T2 active lesions and the proportion of T2 active scans; increases were seen in the proportion of patients with no T2 active lesions [1].

Neutralizing antibodies (NAbs) can occur with any IFN therapy for MS and may impact on efficacy, although there is debate on the degree of this effect [4]. In the EVIDENCE study, 25% of patients receiving IFN beta-1a, 44 mcg sc tiw, and 2% of patients receiving IFN beta-1a, 30 mcg im tiw, had NAb titres greater than 20 neutralizing units/mL at week 48. There was no apparent effect on clinical efficacy for relapse outcomes, but fewer T2 active lesions were seen in the IFN beta-1a, 44 mcg sc tiw, NAb- group compared with the NAb+ group (0.6 versus 1.6 lesions, p = 0.0004).

The aim of this post-hoc analysis of the EVIDENCE data was to establish whether reductions in T2 burden of disease (BOD) were also greater for patients treated with IFN beta-1a, 44 mcg sc tiw, than for those treated with IFN beta-1a, 30 mcg im qw. Whether NAb status affected treatment outcomes was also assessed.

## Methods

### Design and objectives

A post-hoc analysis was performed on MRI data that were collected prospectively from the EVIDENCE study (protocol 21125) [3]. This included all randomized patients who had received at least one dose of the study drug and had evaluable T2-weighted MRI scans from both before the start of dosing (week 0) and at week 48. The central MRI analysis laboratory (USC MS/MRI Research Group) performed the original activity analysis and remained blinded for the current analysis throughout. An experienced MRI radiologist evaluated all MRI scans and remained blinded to treatment. For the T2 BOD analysis, digital data for the proton density/T2 scans for weeks 0 and 48 were used and the radiologist electronically tagged all T2 lesions for segmentation. Trained technicians blinded to study treatment grew each tagged T2 lesion semi-automatically using proprietary software. The radiologist performed a final quality-control step before summarizing the results as a total volume (mm^3^). Combined unique lesion activity (CUA) analysis was performed previously up to week 24 and defined as a Gd-enhancing lesion, new T2 lesion and/or enlarging T2 lesion; this takes into account that a newly active lesion may appear on both the MRI following Gd enhancement and on the T2-weighted MRI, and ensures that it is counted only once. The primary measure of interest was percentage change in BOD (mm^3^) from baseline to week 48. Secondary measures of interest were absolute change in BOD from baseline (week 0) to week 48; percentage and absolute change in BOD from baseline to week 48 when stratified by NAb status (patients with NAb titres ≥ 20 neutralizing units/mL were considered to be NAb+); and the correlation between changes in BOD from baseline to week 48 and changes in CUA from baseline to week 24. NAbs were measured using a cytopathic effect assay [5].

### Statistical analyses

Change in BOD from baseline was compared between treatment groups using an analysis of covariance (ANCOVA) model with effect of treatment and baseline BOD as a single covariate. The treatment difference in means, adjusted for any baseline parameters that were significantly different between the groups, and the associated standard error (SE), were estimated on the raw data from the ANCOVA model. Treatment comparison p values were calculated using a similar ANCOVA model on ranked data. Histograms were constructed by treatment group to show and evaluate the distribution of percentage and absolute change in BOD from baseline to week 48.

Differences in percentage change in BOD between patients receiving IFN beta-1a, 44 mcg sc tiw, and IFN beta-1a, 30 mcg im qw, were calculated by subtracting corresponding adjusted mean changes for each group. A negative value of the difference in changes indicated that treatment with IFN beta-1a, 44 mcg sc tiw, resulted in a larger decrease in BOD than treatment with IFN beta-1a, 30 mcg im qw. Absolute change in BOD was calculated by subtracting the baseline assessment from the post-treatment assessment. A negative value in either the absolute change or percentage change indicated suppression in BOD.

For each treatment group, the relationship between changes in CUA from baseline to week 24 and changes in BOD from baseline to week 48 was assessed using a Spearman's rank correlation analysis.

## Results

A total of 677 patients were randomized to treatment in the EVIDENCE study. Of these, 553 patients met the inclusion criteria for this analysis (IFN beta-1a, 44 mcg sc tiw group: n = 279; IFN beta-1a, 30 mcg im qw group: n = 274). Three of 56 centres that participated in the EVIDENCE study were only able to provide film (not digital) MRI data that was suitable for the activity analysis reported previously [3], but was not suitable for BOD. This affected both treatment groups equally. The two treatment groups did not differ significantly in demographics or baseline lesion characteristics (Table 1).

**Table 1:** Baseline patient demographics and burden of disease.

	**Interferon beta-1a treatment group**		
		
	**44 mcg sc tiw (n = 279)**	**30 mcg im qw (n = 274)**	p value*
Age in years, mean (SD)	38.6 (8.8)	37.7 (8.6)	0.188
Sex, n (%)			0.269
Men	64 (22.9)	74 (27.0)	
Women	215 (77.1)	200 (73.0)	
Race, n (%)			0.935
White	258 (92.5)	250 (91.2)	
Black	12 (4.3)	15 (5.5)	
Asian	0	1 (0.4)	
Hispanic	5 (1.8)	4 (1.5)	
Other	4 (1.4)	4 (1.5)	
BOD in mm^3^, median (range)	5438 (85–135276)	6010 (391–103495)	0.541

*Treatment comparisons with respect to sex and race distributions were performed using a Chi-squared test or a Fisher's Exact test, where appropriate. With respect to age and baseline BOD, an analysis of covariance model using ranked data was performed with effects for treatment.

Numbers do not add to 100% due to rounding.

BOD, burden of disease; im, intramuscular; qw, once weekly; sc, subcutaneous; SD, standard deviation; tiw, three times weekly.

**Figure 1:**
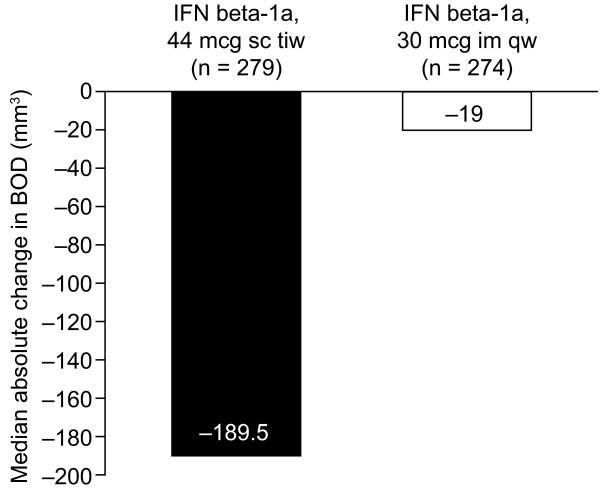
**Median absolute change in burden of disease (BOD) in each treatment group.** IFN, interferon; im, intramuscular; qw, once weekly; sc, subcutaneous; tiw, three times weekly.

**Figure 2:**
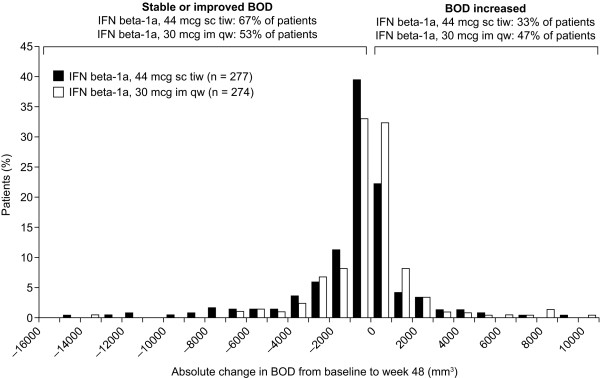
**Distribution of absolute change in burden of disease (BOD) from baseline to week 48 in the two treatment groups.** For presentation purposes, two patients receiving interferon (IFN) beta-1a, 44 mcg subcutaneously (sc) three times weekly (tiw), were removed from this plot; one patient had an extreme increase from baseline value of 56868.5 mm^3 ^and another had an extreme reduction from baseline of 23453.6 mm^3 ^percent changes in BOD. im, intramuscular; qw, once weekly.

**Figure 3:**
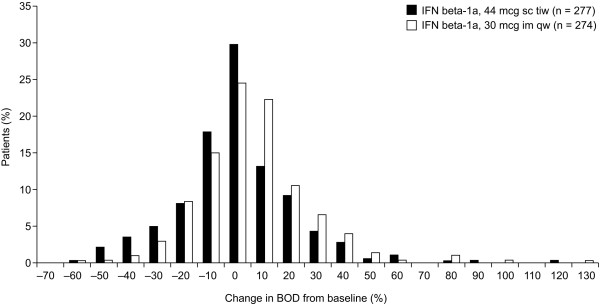
**Distribution of percentage change in burden of disease (BOD) from baseline to week 48 in the two treatment groups.** For presentation purposes, two patients were removed from this plot because they had extreme percentage changes in BOD from baseline to week 48; one patient receiving interferon (IFN) beta-1a, 44 mcg subcutaneously (sc) three times weekly (tiw), had an increase of 431% from baseline and the other patient receiving IFN beta-1a, 30 mcg intramuscularly (im) once weekly (qw), had an increase of 197%.

Median percentage decreases in BOD were greater in the IFN beta-1a, 44 mcg sc tiw, treatment group (-6.7%; range: -65 to 431%) compared with the IFN beta-1a, 30 mcg im qw, treatment group (-0.6%; range: -61 to 197%). The adjusted mean treatment difference (AMTD) in percentage change in BOD from baseline to week 48 showed a significant treatment benefit for patients treated with IFN beta-1a, 44 mcg sc tiw, over those treated with IFN beta-1a, 30 mcg im qw (-4.6%; SE: 2.6%; p = 0.002). Correspondingly, patients in the IFN beta-1a, 44 mcg sc tiw, treatment group had a greater median absolute change in BOD (-189.55 mm^3^; range: -23454 to 56869 mm^3^) compared with those in the IFN beta-1a, 30 mcg im qw, treatment group (-19.0 mm^3^; range: -13337 to 10161 mm^3^; Figure 1). The distribution of absolute and percentage changes in BOD from baseline to week 48 are presented in Figures 2 and 3 and indicate that more patients in the IFN beta-1a, 44 mcg sc tiw, treatment group had greater reductions in BOD compared with the IFN beta-1a, 30 mcg im qw, treatment group. In total, 67% of patients receiving IFN beta-1a, 44 mcg sc tiw, had a stable or improved BOD compared with 53% of patients receiving IFN beta-1a, 30 mcg im qw, and 47% of patients in the IFN beta-1a, 30 mcg im qw, treatment group had increased BOD between weeks 0 and 48 compared with 33% of patients in the IFN beta-1a, 44 mcg sc tiw, treatment group.

**Figure 4:**
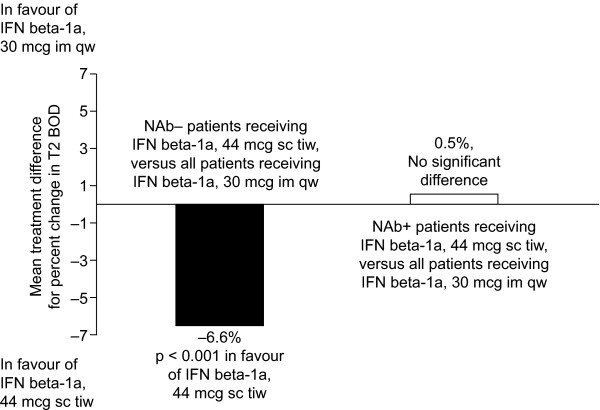
Mean treatment difference for percentage change in T2 burden of disease (BOD) between interferon (IFN) beta-1a, 44 mcg subcutaneously (sc) three times weekly (tiw), neutralizing antibody negative (NAb-), or IFN beta-1a, 44 mcg sc tiw, NAb+, versus IFN beta-1a, 30 mcg intramuscularly (im) once weekly (qw).

Meaningful between-group comparisons by NAb status were not possible, as only seven (2.5%) patients developed NAbs in the IFN beta-1a, 30 mcg im qw, treatment group. Patients receiving IFN beta-1a, 30 mcg im qw, were, therefore, treated as a single group. Median percentage changes in BOD in the IFN beta-1a, 44 mcg sc tiw, NAb+ patients, IFN beta-1a, 44 mcg sc tiw, NAb- patients and IFN beta-1a, 30 mcg im qw, patients were -0.8, -8.0 and -0.6, respectively. Absolute BOD changes were -46.2, -254.6 and -19.0, respectively. There was no evidence of a significant difference in percentage change in BOD from baseline to week 48 between NAb+ patients receiving IFN beta-1a, 44 mcg sc tiw, and those receiving IFN beta-1a, 30 mcg im qw (AMTD: 0.5%; SE: 3.9%; p = 0.583; Figure 4). The AMTD in percentage change in BOD from baseline to week 48 significantly favoured NAb- patients in the IFN beta-1a, 44 mcg sc tiw, group over the IFN beta-1a, 30 mcg im qw, group (-6.6%; SE: 2.8%; p < 0.0001).

A correlation was seen between the change in BOD from baseline to week 48 and change in CUA from baseline to week 24 within the IFN beta-1a, 44 mcg sc tiw, treatment group (*r *= 0.385; p < 0.0001 for absolute and percentage changes) and the IFN beta-1a, 30 mcg im qw, treatment group (*r *= 0.179; p < 0.01 for absolute and percentage changes), respectively.

## Discussion and conclusion

In the current post-hoc analysis, patients with RRMS who were treated with IFN beta-1a, 44 mcg sc tiw, had a greater reduction in T2 BOD after 48 weeks than those given IFN beta-1a, 30 mcg im qw. This finding is consistent with other clinical and MRI outcome measures in the prospective EVIDENCE study. Previous prospectively defined analyses showed that patients receiving IFN beta-1a, 44 mcg sc tiw, also had a reduced likelihood of relapse and had significant reductions in CUA and T2 active lesions compared with those randomized to IFN beta-1a, 30 mcg im qw [1-3].

In general, the impact of both IFNs on suppressing Gd-enhancing lesions on MRI is seen quite early, within the first 1–2 months of initiating therapy [6]. Thus, it was considered reasonable to compare the overall change in T2 BOD for a relatively short duration of observation. Furthermore, from previous experience, looking at BOD differences over 12 months maximizes the group change over the 'noise' created by individual variability. For completeness, the data are presented as a histogram to show the spread of the response for both therapies. In this way it can be seen that both therapies can be associated with a stable MRI outcome or a worsening MRI outcome.

The distribution of absolute and percentage changes in BOD (Figures 2 and 3) in the current analysis indicates that patients in both treatment groups benefited from treatment with IFN beta-1a, but 14% more patients in the IFN beta-1a, 44 mcg sc tiw, treatment group had a stable or improved BOD compared with those treated with IFN beta-1a, 30 mcg im qw. In this 48-week analysis, there was an insufficient number of patients who had a clinical progression in EDSS to explore this relationship further.

A new formulation of IFN beta-1a, 44 mcg sc tiw, has been developed and is currently being studied in a large-scale, Phase III, clinical trial (protocol 25632). Initial data indicate that this new formulation has a reduced immunogenic potential, which should reduce the likelihood of NAb development [7].

It is unclear how much NAbs affect clinical efficacy. NAbs take time to develop and patients with NAbs at 48 weeks may not have had NAbs during most of the preceding time. Likewise, some patients who had developed NAbs earlier may have lost them by week 48. Although some studies suggest that treatment success rates may be curtailed by the development of high titres of persistent NAbs in a minority of patients following long-term treatment with IFN beta [8-10], other studies have shown that the clinical efficacy of IFN beta is the same in patients who are NAb+ as in those who are NAb- [1,2,11]. However, the studies that did not show an effect of NAbs were less than 2 years in duration, and thus may not have been of sufficient duration to show an effect of NAbs on clinical outcomes. In the current analysis, sample sizes were not large enough to allow a direct comparison between NAb+ patients in each treatment group. Still, a comparison of patients receiving IFN beta-1a, 44 mcg sc tiw, who developed NAbs, with all patients receiving IFN beta-1a, 30 mcg im qw (irrespective of NAb status), suggested that NAb+ patients in the IFN beta-1a, 44 mcg sc tiw, treatment group had a similar reduction in T2 BOD to the overall IFN beta-1a, 30 mcg im qw, treatment group. In other words, patients with NAbs who receive high-dose, high-frequency IFN beta still gain similar treatment benefits as those, regardless of NAb status, who receive low-dose, low-frequency IFN beta. This means that, although efficacy may be attenuated with NAbs, therapeutic effect is still provided by IFN beta-1a, 44 mcg sc tiw, on BOD suppression. At week 48, a similar number of T2 active lesions was seen in NAb+ patients in the IFN beta-1a, 44 mcg sc tiw, treatment group (mean 1.6 lesions/patient/scan) as overall (NAb+ and NAb-) in the IFN beta-1a, 30 mcg im qw, treatment group (mean 1.4 lesions/patient/scan). However, the reduction in BOD was smaller in the IFN beta-1a, 44 mcg sc tiw, NAb+ group than in the IFN beta-1a, 44 mcg sc tiw, NAb- group at 48 weeks. This may suggest that MRI is more sensitive than clinical outcomes in showing an early effect of NAbs on efficacy.

This study is probably too short to address adequately a delayed impact of NAbs on MRI outcomes. To some degree, this has been assessed in the PRISMS (Prevention of Relapses and disability by Interferon beta-1a Subcutaneously in Multiple Sclerosis) study, in which results at 48 months showed an increase in BOD in the NAb+ group of 17.6% and a decrease of 8.5% in the NAb- group (p < 0.001) [12].

Interestingly, in the present study there was no apparent impact of NAbs on relapse outcomes at week 48. Thus, these MRI data indicate only a partial impact of NAbs on efficacy outcomes at this time.

## Competing interests

Drs A AL-Sabbagh, R Bennett and P Chang are employed by EMD Serono, Inc. The study was supported by Merck Serono International S.A., Geneva, Switzerland.

## Authors' contributions

AT and DL carried out the MRI design and analysis and interpretation of results. All authors have read and approved the manuscript.

## Pre-publication history

The pre-publication history for this paper can be accessed here:


